# Cryo-EM of α-tubulin isotype-containing microtubules revealed a contracted structure of α4A/β2A microtubules: α4A/β2A microtubules display contracted lattices

**DOI:** 10.3724/abbs.2023130

**Published:** 2023-07-13

**Authors:** Lei Diao, Wei Zheng, Qiaoyu Zhao, Mingyi Liu, Zhenglin Fu, Xu Zhang, Lan Bao, Yao Cong

**Affiliations:** 1 State Key Laboratory of Cell Biology Shanghai Institute of Biochemistry and Cell Biology Center for Excellence in Molecular Cell Science University of Chinese Academy of Sciences Chinese Academy of Sciences Shanghai 200031 China; 2 State Key Laboratory of Molecular Biology Shanghai Institute of Biochemistry and Cell Biology Center for Excellence in Molecular Cell Science University of Chinese Academy of Sciences Chinese Academy of Sciences Shanghai 200031 China; 3 School of Life Science and Technology ShanghaiTech University Shanghai 201210 China; 4 Shanghai Advanced Research Institute Chinese Academy of Sciences Shanghai 201210 China

**Keywords:** microtubule, tubulin isotype, α4A, cryo-EM structure

## Abstract

Microtubules are hollow α/β-tubulin heterodimeric polymers that play critical roles in cells. In vertebrates, both α- and β-tubulins have multiple isotypes encoded by different genes, which are intrinsic factors in regulating microtubule functions. However, the structures of microtubules composed of different tubulin isotypes, especially α-tubulin isotypes, remain largely unknown. Here, we purify recombinant tubulin heterodimers composed of different mouse α-tubulin isotypes, including α1A, α1C and α4A, with the β-tubulin isotype β2A. We further assemble and determine the cryo-electron microscopy (cryo-EM) structures of α1A/β2A, α1C/β2A, and α4A/β2A microtubules. Our structural analysis demonstrates that α4A/β2A microtubules exhibit longitudinal contraction between tubulin interdimers compared with α1A/β2A and α1C/β2A microtubules. Collectively, our findings reveal that α-tubulin isotype composition can tune microtubule structures, and also provide evidence for the “tubulin code” hypothesis.

## Introduction

Microtubules are cytoskeletal filaments that play various important roles in cellular processes, including intracellular transport, cell division, and establishment of cell polarity [ 1 , 2]. They are highly dynamic tubular polymers assembled from α/β-tubulin heterodimers, which are arranged in a head-to-tail manner to form protofilaments, with ~13 protofilaments associating laterally to form a hollow polar tube [3]. Both α- and β-tubulins comprise multiple genes in vertebrates; for example, mice have more than seven α- and eight β-tubulin genes, and each gene encodes a specific tubulin isotype [4]. Distinct α- and β-tubulin isotypes are expressed in different cell types to achieve diversity in microtubule organization and function [5]. The α-tubulin isotype α1A is highly expressed in postmitotic neurons but is decreased in postnatal and adult stages [ 6, 7]. The β-tubulin isotype β3 is specifically expressed in neurons and important for neurite formation [ 8, 9]. Moreover, various mutations in human tubulin genes have been reported to be closely related to diseases, especially neurodevelopmental and neurodegenerative disorders [10], indicating that some tubulin isotypes display unique functions in these processes.

Previous studies have shown that some tubulin isotypes or tubulin constitutions could affect microtubule functions and structures. In mice, β-tubulin isotypes such as β1, β2B, and β4A did not fully rescue the neural migration defects caused by the downregulation of neuronal β3 expression [9] . *In vitro*, purified recombinant α/β3 microtubules had a higher catastrophe frequency than α/β2B microtubules [11]. Cryo-electron microscopy (cryo-EM) studies revealed that the yeast microtubule lattice was expanded compared with mammalian microtubules [12]. Another study on *Caenorhabditis elegans* ( *C*. *elegans*) and *Bos taurus* ( *B*. *taurus*) microtubules showed that in the absence of templates, nucleated *C*. *elegans* microtubules have smaller protofilament numbers than *B*. *taurus* microtubules [13]. Furthermore, it has been suggested that the lateral contact loops are ordered in *C*. *elegans* but unresolved in *B*. *taurus* [13]. Moreover, other studies with a single tubulin isotype purified from insect cells showed that GMPCPP-α1A/β3 microtubules have subtle differences at polymerization interfaces compared with GMPCPP-brain microtubules [14], and GMPCPP-α1B/β2B microtubules are more stable and consist of more protofilaments than α1B/β3 microtubules [5]. Altogether, it appears that microtubules assembled with different tubulin isotypes or tubulin constitutions exhibit distinct microtubule structures. There is also a “tubulin code” hypothesis stating that multiple tubulin isotypes and diverse posttranslational modifications determine microtubule properties and functions [15].

The α/β-tubulin heterodimers are composed of α- and β-tubulin isotypes, and each subunit contains a GTP binding site at the longitudinal interface between the isotypes. The GTP bound to β-tubulin at the E-site (exchangeable) is hydrolysed within the microtubule assembly proceeding [16] via longitudinal contacts with α-tubulin, while the GTP bound to α-tubulin at the N-site (non-exchangeable) plays a structural role and is never hydrolysed [17]. Recently, we reported that α1A/β2A and α1C/β2A form microtubules displaying distinct properties, which was mainly mediated by the C-terminal tail of α-tubulin [18]. However, the effects of specific α-tubulin isotype on microtubule structure remain largely unknown.

In the present study, we examined the structural features of α1A/β2A and α1C/β2A microtubules which exhibit large distinct dynamics [18] as well as α4A/β2A microtubules which possess a special bending feature [19]. By using total internal reflection fluorescence (TIRF) microscopy assay, we showed that α1A/β2A, α1C/β2A and α4A/β2A tubulin dimers were all able to assemble into microtubules in the presence of GMPCPP and Taxol. We determined the cryo-EM structures of the α1A/β2A, α1C/β2A, and α4A/β2A microtubules at the resolution range of 4.2 Å to 4.4 Å. Furthermore, compared with α1A/β2A and α1C/β2A microtubules, α4A/β2A microtubules displayed a longitudinal contraction between tubulin interdimer, suggesting that α-tubulin isotypes are able to tune microtubule structure. This study also expands the current knowledge of α-tubulin isotypes on microtubule functions.

## Materials and Methods

### Plasmid construction

The expression constructs for α1A (NM_011653.2), α1C (NM_009448.4), α4A (NM_009447.4), and β2A (NM_009450.2) were cloned from cDNA of mouse brain tissue into the vector pFastBac Dual. The α1A, α1C, and α4A sequences were inserted after the polyhedron promoter, and the β2A sequence was inserted after the p10 promoter. For affinity purification, a sequence encoding the GGSGG linker and a Flag tag was fused to the 3′ end of the β2A sequence. For α1A, α1C, and α4A, a His tag was inserted in the acetylation loop (between I42 and G43) [20]. An enhancer L21 sequence [21] was added to each of these sequences just before the start codon. The expression construct for kinesin motor domain (KIF5B, NM_004521.3), truncated (amino acids 1–349) and defective in ATP hydrolysis (E236A) ability [Kin _349_(E236A)], was cloned from cDNA of HEK293T cells into vector pET28a with a His tag before the N-terminal of Kin _349_(E236A).

### Expression and purification of mouse recombinant tubulin

Recombinant tubulin was purified by using the Bac-to-Bac system (Life Technologies, Carlsbad, USA) as described previously [18]. Briefly, SF9 cells (Life Technologies) were grown to 2.0–2.5×10 ^6^ cells/mL in Sf-900™ II SFM (10902088; Thermo Fisher, Waltham, USA) supplemented with penicillin and streptomycin, and infected with P3 viral stocks. Cells were cultured in suspension at 27°C and harvested at 72 h after infection. The following steps were performed at 4°C or on ice. Cells were lysed by sonication in lysis buffer (80 mM PIPES, pH 6.9, 100 mM KCl, 1 mM MgCl _2_, 1 mM EGTA, 0.1 mM GTP, 0.5 mM ATP, and 1 mM PMSF), and then the lysate was centrifuged for 30 min at 35,000 *g*. The supernatant was filtered through a 0.45-μm Millex-HV PVDF membrane (Millipore, Billerica, USA) and loaded on a nickel-nitrilotriacetic acid column (Qiagen, Hilden, Germany) pre-equilibrated with lysis buffer. The column was washed with 30 mL of wash buffer (lysis buffer supplemented with 25 mM imidazole) and then eluted with elution buffer [1× BRB80 (80 mM PIPES, 1 mM MgCl _2_, and 1 mM EGTA), 300 mM imidazole, and 0.1 mM GTP, pH 7.0]. The eluate was diluted with an equal volume of BRB80 buffer supplemented with 0.1 mM GTP and mixed with anti-Flag antibody-conjugated resin (Sigma-Aldrich, St Louis, USA) for 2 h. Flag-tagged tubulin was eluted with BRB80 buffer supplemented with 0.2 mg/mL 3× Flag peptide (A6001; APE×BIO, Houston, USA). Finally, the purified tubulin was concentrated and desalted on an Amicon Ultracel-30 K filter (Millipore) with BRB80 and 0.1 mM GTP. The tubulin concentration was estimated by measuring the UV absorbance at 280 nm, and tubulin samples were frozen in liquid nitrogen and stored at –80°C.

### Purification of Kin _349_(E236A)

The Kin _349_(E236A) was expressed and purified from *Escherichia coli* ( *E*. *coli*) BL21 (DE3) using a protocol that was modified from previously published method [22]. The transformed *E* . *coli* BL21 (DE3) cells were cultured in Luria-Bertani medium (LB) at 37°C until the OD _600 nm_ was between 0.6 and 0.8, and then 0.5 mM isopropyl-β-D-thiogalactopyranoside (IPTG; Sigma-Aldrich) was added for the induction of protein expression at 20°C for 16–18 h. Cells were harvested and resuspended in lysis buffer (20 mM PIPES pH 6.9, 150 mM KCl, 4 mM MgCl _2_, 0.1 mM ATP, and 1 mM PMSF) and lysed by sonication. The lysate was cleared by centrifugation at 35,000 *g* for 20 min at 4°C. The supernatant was filtered through a 0.45-μm Millex-HV PVDF membrane and loaded on a nickel-nitrilotriacetic acid column pre-equilibrated with lysis buffer. The column was washed with 30 mL of wash buffer (lysis buffer supplemented with 25 mM imidazole) and eluted with elution buffer (80 mM PIPES pH 6.9, 4 mM MgCl _2_, 1 mM EGTA, 300 mM imidazole, and 0.1 mM ATP). The proteins were concentrated and gel-filtrated on a SuperdexTM 75 size exclusion chromatography column (GE Healthcare, Madison, USA) equilibrated with stock buffer (80 mM PIPES pH 6.9, 100 mM KCl, 4 mM MgCl _2_, 1 mM EGTA, and 0.1 mM ATP). Protein fractions containing the target protein were collected and concentrated. The protein concentration was estimated by measuring the UV absorbance at 280 nm. Protein samples were frozen in liquid nitrogen and stored at ‒80°C.

### Microtubule preparation for the TIRF assay

For GMPCPP- or Taxol-stabilized α1A/β2A, α1C/β2A, and α4A/β2A microtubules, 7.5 μM α1A/β2A, α1C/β2A, or α4A/β2A tubulins, including ~4% biotin-tubulin (T333P, Cytoskeleton, Denver, USA) and ~4% rhodamine-tubulin (TL590M; Cytoskeleton), were incubated with 1 mM GMPCPP or 1 mM GTP with 10 mM Taxol at 37°C for 3 h. Then, the solution was diluted in warmed BRB80 (for GMPCPP microtubules) or BRB80 containing 1 mM GTP and 10 mM Taxol (for Taxol microtubules) and incubated in cleaning and silanizing glass coverslips for 5 min. Images were acquired by TIRF microscopy with a cell observer spinning disk system (Zeiss, Oberkochen, Germany) and a 100× oil lens. The images were recorded with a pixel size of 160 nm.

### Formation of kinesin-decorated α1A/β2A, α1C/β2A, and α4A/β2A microtubules

For the construction of GMPCPP-stabilized α1A/β2A, α1C/β2A, and α4A/β2A microtubules, 15‒20 μM α1A/β2A, α1C/β2A, or α4A/β2A tubulins was incubated with 1 mM GMPCPP at 37°C for 3 h. To remove unpolymerized tubulin, the solution was centrifuged at 126,000 *g* for 5 min at 27°C, and the supernatant was discarded. Then, the microtubule pellet was resuspended in cold BRB80 and depolymerized at 4°C for 20 min, followed by a second round of polymerization at 37°C with 1 mM GMPCPP for 3 h. GMPCPP-stabilized microtubules were pelleted as described above and resuspended in 37°C pre-warmed BRB80 with ~80% volume of the initial reaction. The microtubule solution was diluted at a ratio of 1:2‒1:3, mixed with ~20 μM Kin _349_(E236A) and 2 mM ATP, and then incubated for 5 min at room temperature.

### Cryo-EM sample preparation

To prepare the cryo-EM sample of the kinesin-decorated α1A/β2A microtubules, an aliquot of 2.2 μL sample was applied to a plasma-cleaned holey carbon grid (Quantifoil R1.2/1.3, Cu, 400 mesh). The grid was blotted with a Vitrobot Mark IV (Thermo Fisher Scientific) using a blot force of –1 and 1 s blot time at 100% humidity and 22°C and then plunged into liquid ethane cooled by liquid nitrogen. For cryo-EM sample preparation of the kinesin-decorated α1C/β2A or α4A/β2A microtubules, a similar procedure was applied.

### Cryo-EM data collection

Cryo-EM movies of the samples were collected on a Titan Krios transmission electron microscope (Thermo Fisher Scientific) operated at an accelerating voltage of 300 kV. All movies were recorded on a K2 Summit direct electron detector (Gatan, Pleasanton, USA) operated in the super resolution mode at a nominal magnification of 18,000× (yielding a pixel size of 1.3 Å after 2 times binning) under low-dose condition [23]. Each movie was dose-fractioned into 38 frames with a dose rate of 8 e ^–^ per pixel per second on the detector. The exposure time was 7.6 s with 0.2 s for each frame, generating a total dose of 36 e ^–^/Å ^2^. Defocus values varied from –0.8 to –1.5 μm. All images were collected by utilizing the SerialEM automated data collection software package [23].

### Image processing and 3D reconstruction

For each dataset, motion correction of the image stack was performed using MotionCor2 [24], and CTF parameters were determined using CTFFIND4 [25]. The subsequent image processing for microtubule reconstruction was performed mainly following the previously established method and related procedures [3]. Briefly, the EMAN1 program *helixboxer* was used to select microtubules [26]. Subsequently, the microtubules were divided into overlapping microtubule “particles”, with the box size of 512 pixels and the nonoverlapping distance of adjacent microtubule particles set to ~80 Å. Multireference alignment (MRA) in EMAN1 was used to determine the initial alignment parameters and protofilament number [26]. The microtubule particles with the same protofilament number were merged and subjected to further refinement in FREALIGN v9 [27]. The number of microtubule protofilaments formed *in vitro* varies from 9 to 16, with the majority consisting of 14 protofilaments [28]. Indeed, over 55% of the microtubules contained 14 protofilaments in our assay. Thus, only the dominant 14-protofilament microtubule densities were used for further refinement, with a seam-search protocol described previously [29]. The final resolution for each reconstruction was estimated based on the gold-standard criterion using a Fourier shell correlation (FSC) of 0.143. All of the reconstructions were postprocessed through deepEMhancer [30], and the reconstructions were sharpened by applying a b-factor value of –100 Å ^2^. UCSF Chimera and ChimeraX were applied for structural visualization and figure generation [31] .

### Atomic model building and analysis

Initial models of α1A/β2A, α1C/β2A, and α4A/β2A microtubules were generated through the SWISS-MODEL server [32] using an existing GMPCPP-bound *Chlamydomonas reinhardtii* tubulin model (PDB 6U42) as the template. For the seam region, 16 adjacent tubulin dimer models were fit into the corresponding region of the map in Chimera by rigid-body fitting, which were then refined against the corresponding map by applying *phenix*. *real_space_refine* in Phenix [33] to ensure that the model of the central 4 dimers matches well with the density map. For the non-seam region, 9 dimer models were used to perform the model refinement following a similar procedure to ensure that the model of the central dimer matches well with the density map. All RMSD calculations were performed using UCSF Chimera [31].

## Results

### Recombinant mouse α1A/β2A, α1C/β2A, and α4A/β2A form microtubules

**Figure 1:**
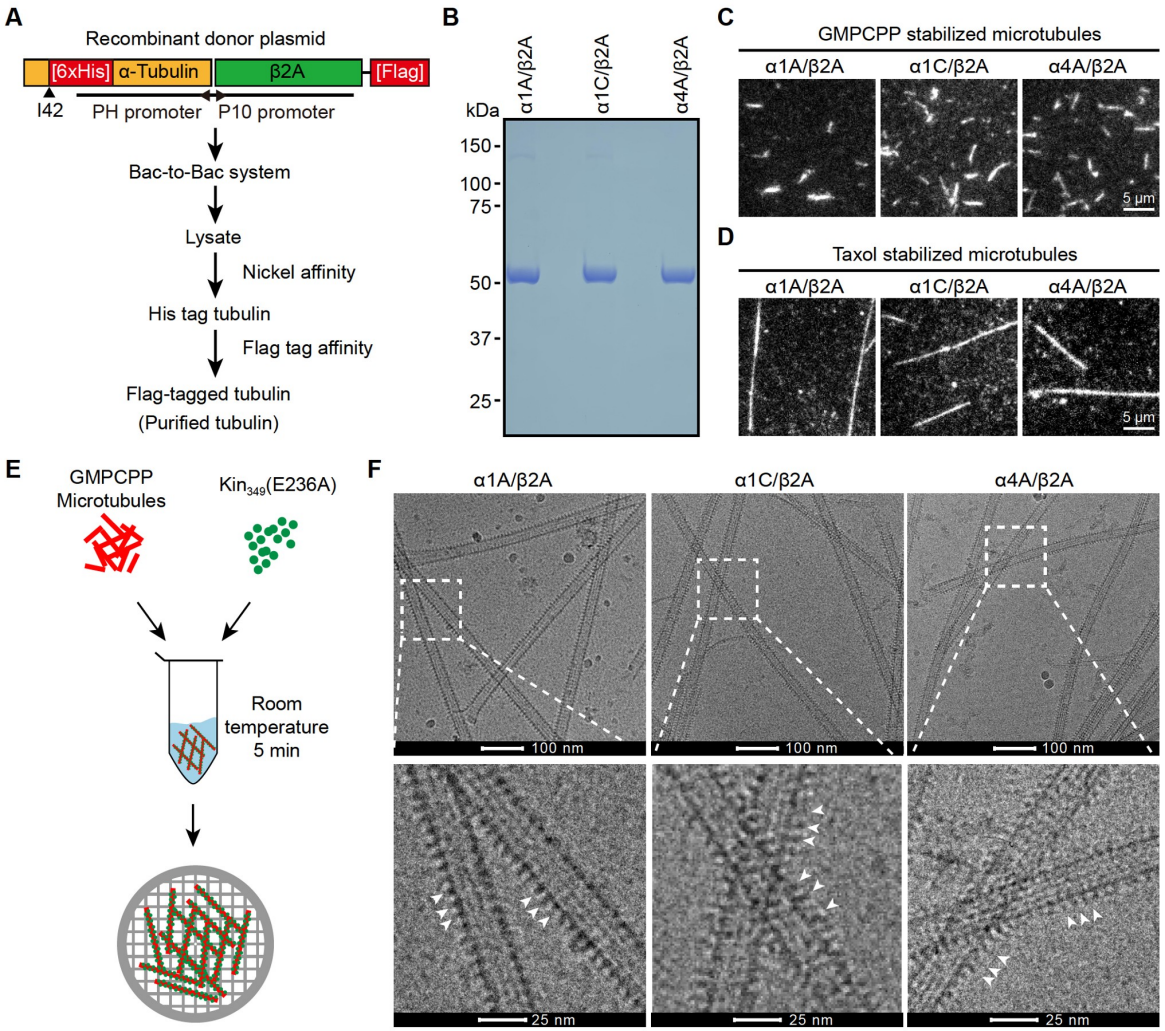
Figure 1 Purification of recombinant mouse α/β-tubulin dimmers (A) Schematic diagram for purification of recombinant tubulin isotypes. (B) Coomassie blue staining of purified α1A/β2A, α1C/β2A, and α4A/β2A. (C,D) TIRF images of GMPCPP (C)- and Taxol (D)-stabilized α1A/β2A, α1C/β2A, and α4A/β2A microtubules. Rhodamine-labelled porcine tubulin (~4%) was added to visualize microtubules, and biotin-labelled porcine tubulin (~4%) was added to immobilize microtubules on neutravidin-coated coverslips. (E) Schematic for cryo-EM sample preparation of α1A/β2A, α1C/β2A, and α4A/β2A microtubules decorated with Kin349(E236A). (F) Representative cryo-EM images of α1A/β2A, α1C/β2A, and α4A/β2A microtubules decorated with Kin349(E236A) (indicated by white arrowhead).

Our recent work on α1A/β2A, α1C/β2A and α4A/β2A microtubules suggested that microtubule polymerization properties and morphologies could be affected by specific α-tubulin isotypes [ 18, 19 ], although the amino acid sequences of these α-tubulin isotypes are highly conserved ( Supplementary Figure S1). To further explore the effect of α-tubulin isotypes on microtubule structures, we expressed and purified different α-tubulin isotypes, including α1A/β2A, α1C/β2A, and α4A/β2A, using a previously established method ( Figure 1A,B) [18]. TIRF assay showed that α1A/β2A, α1C/β2A, and α4A/β2A tubulin dimers were all able to assemble into microtubules in the presence of GMPCPP or Taxol ( Figure 1C,D). Moreover, direct visualization of the cryo-EM images showed that these three types of tubulin dimers could all form microtubules decorated with the kinesin mutant K _349_(E236A) ( Figure 1E,F). These results suggest that tubulin dimers composed of an α-tubulin isotype (including α1A, α1C and α4A) and a constant β-tubulin β2A are able to form microtubules.

### Cryo-EM structures of α1A/β2A, α1C/β2A, and α4A/β2A microtubules

**Figure 2:**
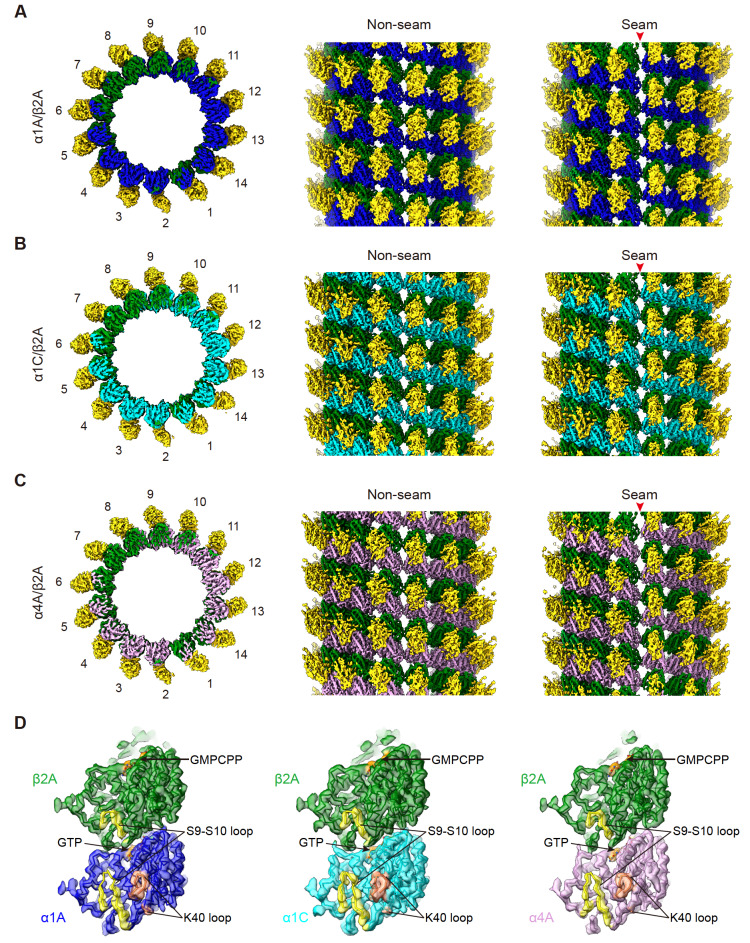
Figure 2 Cryo-EM structures of α1A/β2A, α1C/β2A, and α4A/β2A microtubules (A–C) Overview of the cryo-EM maps for α1A/β2A (A), α1C/β2A (B), and α4A/β2A (C) microtubules decorated with kinesin motor domain, respectively. Kinesin is in gold, β2A in green, α1A in blue, α1C in cyan, and α4A in pink, and this color scheme is followed throughout. Viewed from the top and side of microtubules, respectively. (D) Model and map (transparent surface) fitting of α1A/β2A, α1C/β2A, and α4A/β2A microtubules. Viewed from the lumen side with the key structural elements indicated.

**Figure 3:**
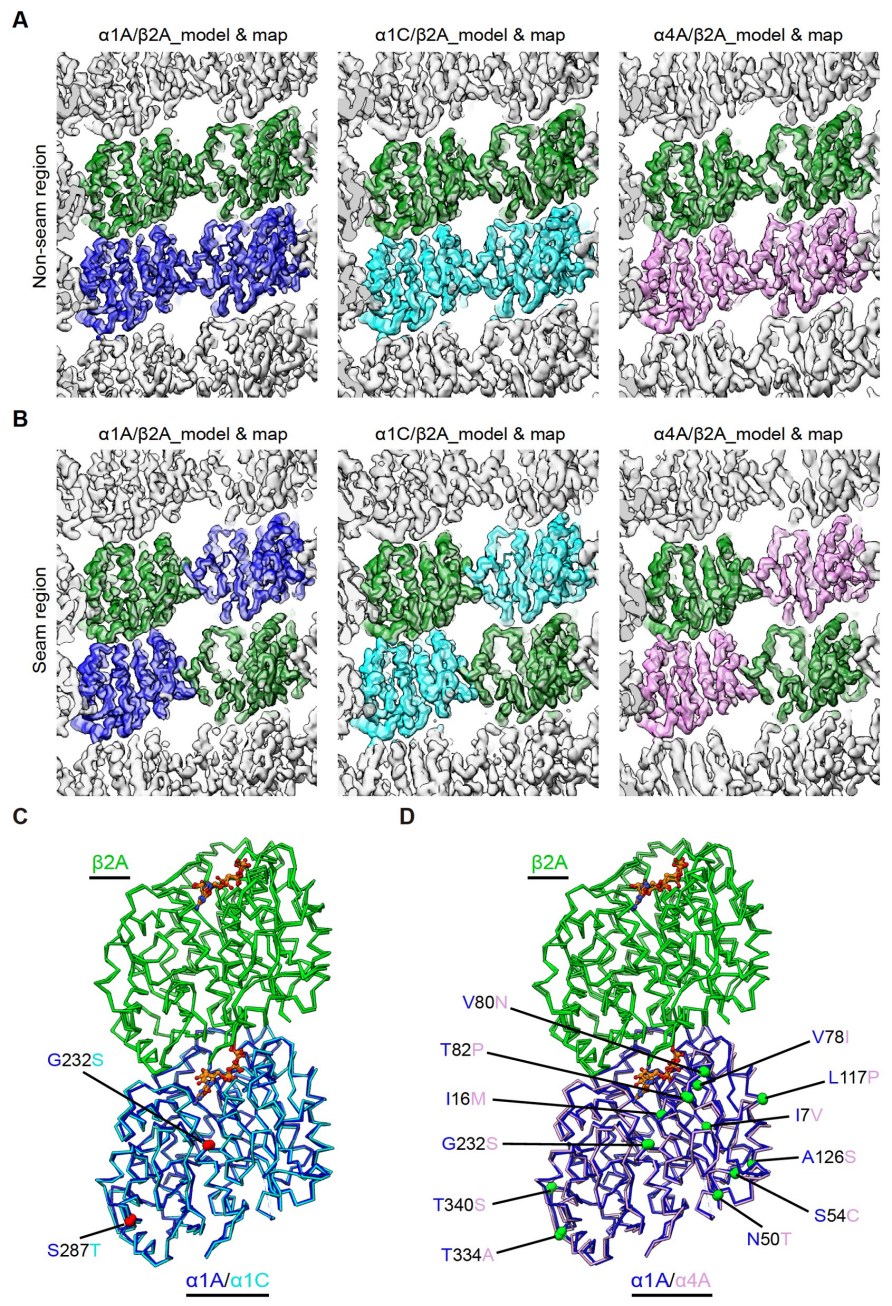
Figure 3 Cryo-EM density maps and models of α1A/β2A, α1C/β2A, and α4A/β2A microtubules (A,B) Model and map fitting of α1A/β2A, α1C/β2A, and α4A/β2A microtubules, showing homotypic lateral interactions at the non-seam region (A) and at the seam region (B), viewed from the lumen side. (C) Comparison of the Cα trace for tubulin dimers between α1A/β2A and α1C/β2A microtubules. The distinct amino acids between α1A and α1C are highlighted in red balls. (D) Comparison of the Cα trace for tubulin dimers between α1A/β2A and α4A/β2A microtubules. The distinct amino acids between α1A and α4A are highlighted in green balls. It appears that there are more amino acids variations between α4A and α1A (D) than between α1C and α1A (C).

To investigate the architectures of α1A/β2A, α1C/β2A, and α4A/β2A microtubules and explore the impact of α-tubulin isotypes on the structural features of the microtubules, we characterized the structures of the three kinds of microtubules in the presence of GMPCPP by using cryo-EM. The microtubules were decorated with K _349_(E236A) to aid in the identification of α- and β-tubulins in the reconstruction process through the “seam-search” protocol ( Figure 1E,F) [ 3, 29]. We determined the cryo-EM maps of α1A/β2A, α1C/β2A, and α4A/β2A microtubules in the dominantly populated 14-protofilament type at the resolution range of 4.2 Å to 4.4 Å ( Figure 2A–C, Supplementary Figure S2 and Supplementary Table S1). We then built the corresponding atomic model for each of the maps, including the non-seam region and the seam region ( Figure 2D and Figure 3 A,B).

Both α- and β-tubulin isotypes contain an S9-S10 loop, which has eight residues longer in α-tubulin than in β-tubulin and forms part of the Taxol-binding site in β-tubulin [ 34, 35]. Here, in our α1A/β2A, α1C/β2A, and α4A/β2A structures, it appears that the S9-S10 loop in α-tubulin is indeed longer than that in β-tubulin ( Figure 2D), demonstrating successful separation of α- and β-tubulins in these reconstructions. Nevertheless, the density of the α-tubulin acetylation loop P37-G45 (also known as the K40 loop) and the C-terminal tail of both α- and β-tubulins were not observed in the maps ( Figure 2D), likely due to the well-known flexibility of these regions [35].

### Similar lateral contacts among α1A/β2A, α1C/β2A, and α4A/β2A microtubules

**Figure 4:**
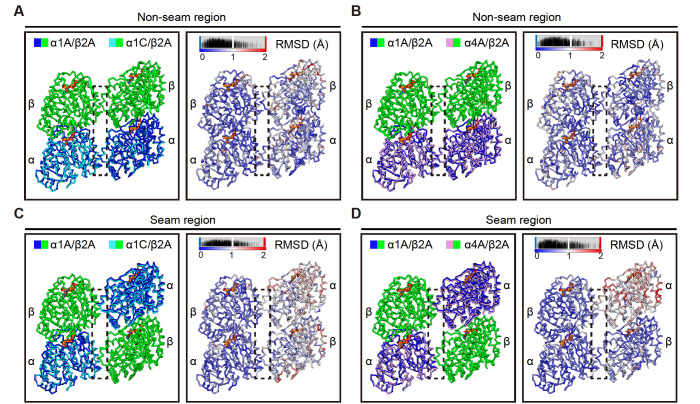
Figure 4 The lateral interactions are similar among α1A/β2A, α1C/β2A, and α4A/β2A microtubules (A,B) Visualization of the lateral interactions in the non-seam region by comparison of the atomic models between α1A/β2A and α1C/β2A microtubules (A) and between α1A/β2A and α4A/β2A microtubules (B) and corresponding Cα atom RMSDs. (C,D) Visualization of the lateral interactions in the seam region by comparison of the atomic models between α1A/β2A and α1C/β2A microtubules (C) and between α1A/β2A and α4A/β2A microtubules (D) and corresponding Cα atom RMSDs. Viewed from the lumen side. The lateral interaction interface between the neighbouring protofilaments is indicated by the dotted black frame.

To understand the effects of α-tubulin on microtubule structure, we first analyzed the structures of α1A/β2A, α1C/β2A, and α4A/β2A dimers and found that these dimers exhibit overall similar conformation ( Figure 3C,D). We then compared the models of two tubulin dimers within adjacent protofilaments in the three obtained microtubule structures. By superimposing the global Cα of α-tubulin on the left side, we found that for α1C/β2A microtubules, the lateral interaction between α-α and β-β contacts in the non-seam region was similar to that of α1A/β2A contacts, with the Cα atom root mean square deviation (RMSD) lower than 1 Å ( Figure 4A). This was also the case for α4A/β2A and α1A/β2A microtubules ( Figure 4B). Similar analysis of the lateral interaction between α-β and β-α contacts in the seam region suggested that the α1C/β2A microtubules display a subtle longitudinally contracted divergence between α1C and α1A in the seam region ( Figure 4C); the α4A/β2A microtubules also display a slight longitudinal contraction (Cα atom RMSD of ~1 Å) between α4A and α1A in the seam region ( Figure 4D). Additionally, we did not observe obvious structural deviation in the lateral interfaces of these three types of microtubules ( Figure 4). Taken together, our data suggest that these α-tubulin isotypes do not show obvious lateral effects on their microtubule structures, while α1C/β2A and α4A/β2A microtubules exhibit slight longitudinal contraction between the interdimers in the seam region.

### A distinct contracted lattice of α4A/β2A microtubule

**Figure 5:**
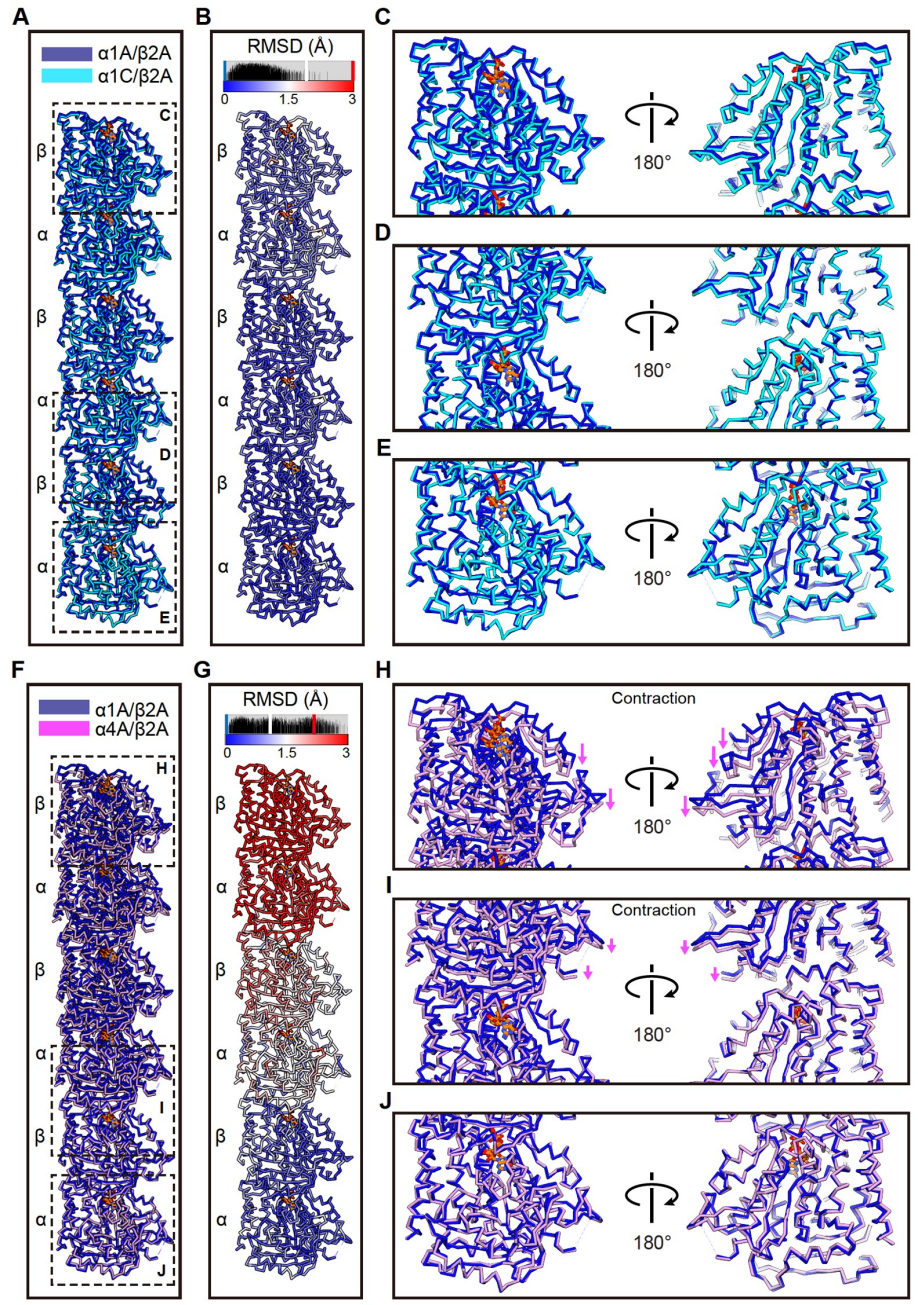
Figure 5 α4A/β2A microtubules display longitudinal contracted lattices (A) Comparison of the Cα trace of three consecutive tubulin dimers between α1A/β2A and α1C/β2A microtubules, superimposed on the bottom α-tubulin. (B) Cα atom RMSDs between the two models shown in (A), with deviations colored from blue to red. (C‒E) Zoom-in view of the boxed regions in (A). Viewed from the lumen side. The rendering style was followed throughout this figure. (F) Comparison of the Cα trace of three consecutive tubulin dimers between α1A/β2A and α4A/β2A microtubules, superimposed on the bottom α-tubulin. (G) Cα atom RMSDs between the two models shown in (F). (H‒J) Zoom-in view of the boxed regions in (F).

Due to the longitudinally contracted divergence in the seam region, especially between α1A/β2A and α4A/β2A microtubules ( Figure 4D), we further compared the structures of α1A/β2A and α1C/β2A and those of α1A/β2A and α4A/β2A in the longitudinal orientation. We first superimposed three consecutive tubulin dimers within a single protofilament of α1A/β2A and α1C/β2A microtubules by aligning the Cα of the bottom α-tubulin together. We found that the longitudinal interactions between the intradimer and interdimer of α1A/β2A and α1C/β2A microtubules are very similar ( Figure 5A–E), which was confirmed by Cα atom RMSD analysis ( Figure 5B). Surprisingly, similar analysis of α1A/β2A and α4A/β2A microtubules revealed a clear contraction of α4A/β2A relative to the α1A/β2A protofilament ( Figure 5F–J). This contraction mainly exists between the longitudinal interdimer, consistent with that observed in the seam region ( Figure 4D). Additionally, this contraction is in a progressive expanded manner that increases with each dimer along the protofilament, such that the first neighbouring α4A/β2A dimer contracts by ~1.5 Å relative to α1A/β2A microtubules, then the second dimer contracts by 3 Å, and so on. Cα atom RMSD analysis also confirmed these contraction divergences ( Figure 5G). Collectively, our structural analysis revealed that α4A/β2A microtubules displayed a longitudinal contraction between tubulin interdimer compared with α1A/β2A and α1C/β2A microtubules, indicating that microtubule structures can be regulated by different α-tubulin isotypes.

## Discussion

Microtubules assembled from different tubulin constitutions or with different tubulin isotypes exhibit distinct structures. For instance, previous cryo-EM studies revealed that microtubules assembled from different sources, such as yeast, porcine brain, *C*. *elegans* or *B*. *taurus* brain, exhibit different structures [ 12, 13, 36]. Other cryo-EM studies with single tubulin isotypes showed that GMPCPP-α1A/β3 and GMPCPP-brain microtubules display different structures [14], which is also the case for GMPCPP-α1B/β2B and GMPCPP-α1B/β3 microtubules [5]. In the present study, we assembled and determined the cryo-EM structures of α1A/β2A, α1C/β2A, and α4A/β2A microtubules, which were not previously available. Our structural analysis revealed that, compared with α1A/β2A and α1C/β2A microtubules, α4A/β2A microtubules exhibit longitudinal contraction between tubulin interdimers, which could potentially be related to more amino acid variations between α4A and α1A relative to that between α1C and α1A ( Figure 3C,D and Supplementary Figure S1). Collectively, our results suggest that α-tubulin isotypes can tune microtubule structure.

Microtubules are dynamic polymers of α/β-tubulin that undergo polymerization and depolymerization, which are essential for their functions. Previous studies have suggested that some tubulin isotypes have a distinct influence on the dynamic properties of microtubules; for instance, microtubules composed of α1B/(β1+β4B) possess a faster growth rate and lower catastrophe frequency [37], α/β3 microtubules display a higher catastrophe frequency than α/β2B microtubules [11], and α1C/β2A microtubules exhibit a faster growth rate and higher catastrophe frequency than α1A/β2A microtubules [18]. In the present study, we assembled α1A/β2A and α1C/β2A microtubules, determined their cryo-EM structures, and found that they displayed similar structural features. However, the tubulin C-terminal tails that extend from the microtubule surface were unresolved, most likely due to their flexibility. This feature, to some extent, confirmed our previous conclusion that the distinct dynamics of α1A/β2A and α1C/β2A microtubules is mainly determined by their C-terminal tails [18]. Moreover, our recent study showed that α4A/β2A microtubules had different microtubule polymerization properties and special curved growth features [19]. Furthermore, we assembled and determined the cryo-EM structure of the α4A/β2A microtubule and found that it bore a longitudinal contraction compared with the α1A/β2A and α1C/β2A microtubules. Since GTP hydrolysis can alter the conformation of microtubules [3], whether the contracted structure of GMPCPP-stabilized α4A/β2A microtubules is resulted from its curved growth in the presence of GTP and whether Met16 of α4A mainly determining the curved growth of α4A/β2A microtubules [19] is the key residue that causes its longitudinal contraction remain to be further examined.

Eukaryotic cells usually contain multiple α- and β-tubulin isotypes, and their expression patterns vary widely among different tissues and during different developmental stages [ 38, 39]. The tubulin isotype α4A is highly expressed in various tissues, such as the heart, brain and platelets [ 40, 41], and is mainly expressed at later stages of development [42]. Previous studies suggested that some mutations in tubulin α4A may cause serious diseases, including amyotrophic lateral sclerosis (ALS) [ 43– 45], frontotemporal dementia (FTD) [46], and macrothrombocytopenia [41]. Although all of these original residues before mutations identified thus far are conserved within most of the α-tubulin isotypes, the mutations are only found in α4A, indicating that these mutant amino acids in conjunction with other unique isotype residues together affect microtubule structure and function, and eventually result in diseases. For example, the D417H and R262H mutations in β3, identified in patients with severe congenital fibrosis of extraocular muscle type 3 (CFEOM3), not only impaired the binding of kinesin but also altered microtubule dynamics [ 47– 49]. A recent report showed that α4A played important roles in maintaining the number and arrangement of microtubule coils in the platelet marginal band [41]. We then postulated that the special contracted structure and curved growth feature [19] of α4A/β2A microtubules caused by α4A may favor maintaining the bent microtubules in the platelet marginal band. Moreover, it has been reported that tubulin isotype β2 is predominantly present in the neurites of differentiated neuroblastoma SK-N-SH cells and plays a key role in neurite outgrowth, while both β1 and β3 occur in cell bodies and neurites [50]. However, the distribution of most tubulin isotypes in cells remains largely unclear, mainly due to the lack of specific antibodies to distinguish these highly conserved isotypes.

In summary, we found that α4A/β2A microtubules display a contracted structure, which provides powerful evidence that different tubulin isotype constitutions could alter the structural properties of microtubules. Collectively, our previous [ 18, 19] and current studies demonstrate that α-tubulin isotype composition can tune microtubule dynamics, morphology and structure. Nevertheless, the functional role of specific tubulin isotypes, such as α4A, in cells remains to be further explored.

## Data Availability

All data presented in this study are available within the figures and the supplementary information. Cryo-EM maps have been deposited in the EMDB, and the associated models have been deposited in the PDB: α1A/β2A microtubules (EMD-35790, PDB: 8IXA for the non-seam region, PDB: 8IXB for the seam region), α1C/β2A microtubules (EMD-35791, PDB: 8IXD for the non-seam region, PDB: 8IXE for the seam region), and α4A/β2A microtubules (EMD-35792, PDB: 8IXF for the non-seam region, PDB: 8IXG for the seam region).

## Supporting information

23105Supplementary_data

## Supplementary Data

Supplementary data is available at *Acta* *Biochimica et Biophysica Sinica* online.
