# Supplementary material for: Cryo-EM of α-tubulin isotype-containing microtubules revealed a contracted structure of α4A/β2A microtubules: α4A/β2A microtubules display contracted lattices
Source: Acta Biochim Biophys Sin (Shanghai). 2023 Jul 13;55(10):1551–60. doi: 10.3724/abbs.2023130 (PMC10577476; doi:10.3724/abbs.2023130)
Supplement: 23105Supplementary_data [file 23105Supplementary_data.pdf]

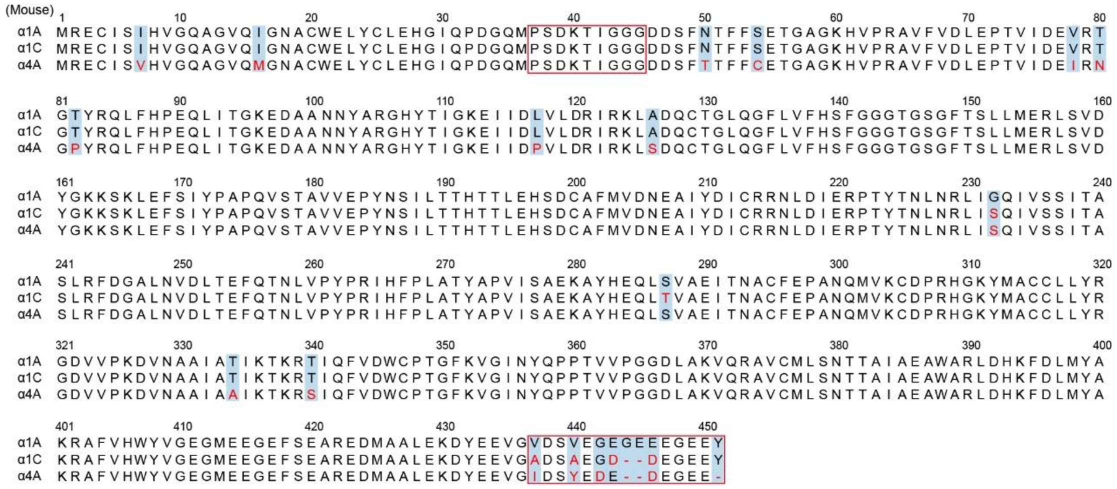

Supplementary Figure S1. Sequence alignment of mouse α1A, α1C and α4A The distinct amino acids are marked in red color font and light blue background. The two flexible regions of α-tubulin, K40 loop and C-terminal tail, are marked in red box.

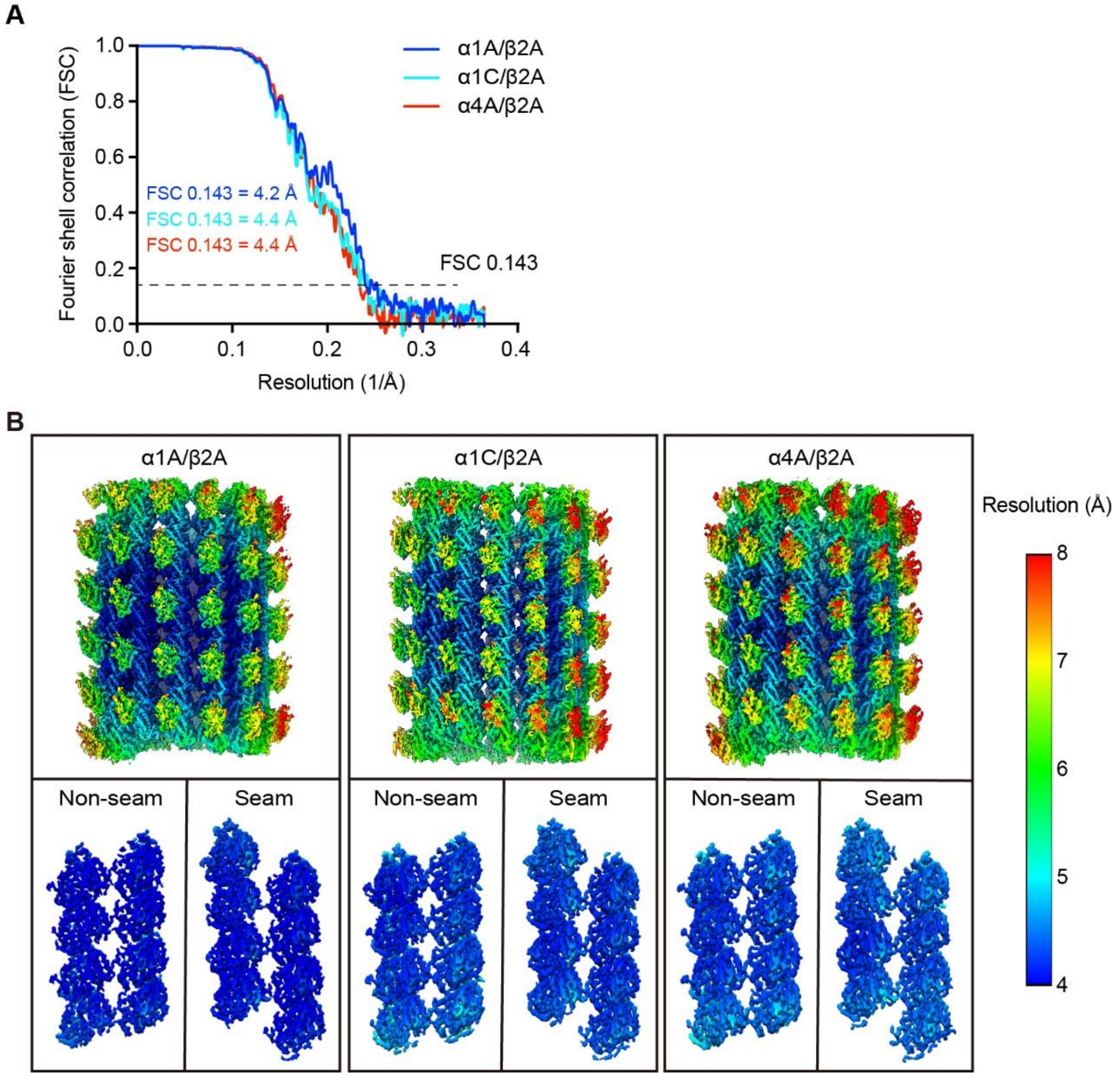

Supplementary Figure S2. Cryo-EM reconstructions of GMPCPP-stabilized α1A/β2A, α1C/β2A, and α4A/β2A microtubules decorated with Kin$_{349}$(E236A) (A) Resolution estimation of the cryo-EM maps for α1A/β2A, α1C/β2A, and α4A/β2A microtubules according to the gold-standard FSC criterion of 0.143. (B) Local resolution estimation of the α1A/β2A, α1C/β2A, and α4A/β2A microtubule maps, with their nonseam and seam regions also displayed from the lumen side. The resolution color bar is shown on the right.

Supplementary Table S1. Cryo-EM data collection and refinement statistics

**Sample**	**α1A/β2A**	**α1C/β2A**	**α4A/β2A**
**Data collection**			
EM equipment	Titan Krios	Titan Krios	Titan Krios
Voltage (kV)	300	300	300
Detector	K2 Summit	K2 Summit	K2 Summit
Pixel size (Å)	1.3	1.3	1.3
Total electron dose ($e^-/Å^2$)	36	36	36
Dose rate (e^-/physical pixel/s)	8	8	8
Exposure time (s)	7.6	7.6	7.6
Defocus range (μm)	-0.8 ~ -1.5	-0.8 ~ -1.5	-0.8 ~ -1.5
**Reconstruction**			
Software	EMAN & Frealign	EMAN & Frealign	EMAN & Frealign
Original particles	32,977	31,952	33,817
Final particles (14 protofilaments)	19,287	21,250	24,668
Final particles (13 protofilaments)	7,068	5,228	4,845
Symmetry	Pseudo-Helix	Pseudo-Helix	Pseudo-Helix
Final resolution (Å)	4.2	4.4	4.4
Twist (°)	-25.75	-25.75	-25.78
Rise (Å)	8.72	8.72	8.62
Map sharpening B-factor	-100	-100	-100
**Atomic modeling (Seam)**			
Software	SWISS-MODEL & Phenix	SWISS-MODEL & Phenix	SWISS-MODEL & Phenix
Model composition			
Atoms	37,380	37,384	37,376
Protein residues	4,712	4,712	4,712
Ligands	12	12	12
Rms deviation			
Bond length (Å)	0.008	0.008	0.008
Bond angle (°)	1.23	0.88	1.23
Ramachandran plot			
Favored (%)	97.12	97.48	96.99
Allowed (%)	2.88	2.52	3.01
Outliers (%)	0.00	0.00	0.00
Clash score	3.06	3.98	3.70
**Atomic modeling (Non-seam)**			
Model composition			
Atoms	84,105	84,114	84,105
Protein residues	10,602	10,602	10,602
Ligands	27	27	27
Rms deviation			
Bond length (Å)	0.008	0.008	0.008
Bond angle (°)	1.22	1.23	1.23
Ramachandran plot			
Favored (%)	97.44	97.76	97.92
Allowed (%)	2.56	2.24	2.08
Outliers (%)	0.00	0.00	0.00

Clash score	3.85	3.38	3.56
